# A demonstration study comparing “role-emergent” versus “role-established” pharmacy clinical placement experiences in long-term care facilities

**DOI:** 10.1186/1472-6920-13-104

**Published:** 2013-08-05

**Authors:** Rosemin Kassam, Mona Kwong, John B Collins

**Affiliations:** 1 Faculty of Medicine, School of Population and Public Health, University of British Columbia, 2206 East Mall, Vancouver, BC V6T 1Z3, Canada; 2 Howe Street Pharmacy, 1070 Howe Street, Vancouver, BC V6Z 1P5, Canada; 3 Department of Educational Studies, University of British Columbia, 2044 Lower Mall, Vancouver, BC V6T 1Z2, Canada

**Keywords:** Residential care, Long-term care, Pharmacy, Clerkship, Clinical education, Clinical practice, Non-traditional clinical placements, Role-emergent, Role-emerging, Institutional, Experiential

## Abstract

**Background:**

Increasing challenges to recruit hospital sites with full-time on-site pharmacy preceptors for institutional-based Advanced Pharmacy Practice Experiences (APPE) has made it necessary to consider alternate experiential models. Sites with on-site discipline specific preceptors to supervise students have typically been referred to in the literature as “role-established” sites. In British Columbia, long-term care (LTC) facilities offered a unique opportunity to address placement capacity issues. However, since the majority of these facilities are serviced by off-site community pharmacists, this study was undertaken to explore the viability of supervising pharmacy students remotely – a model referred to in the literature as “role-emergent” placements. This paper’s objectives are to discuss pharmacy preceptors’ and LTC non-pharmacist staff experiences with this model.

**Methods:**

The study consisted of three phases: (1) the development phase which included delivery of a training program to create a pool of potential LTC preceptors, (2) an evaluation phase to test the viability of the LTC role-emergent model with seven pharmacists (two role-established and five role-emergent) together with their LTC staff, and (3) expansion of LTC role-emergent sites to build capacity. Both qualitative and quantitative methods were used to obtain feedback from pharmacists and staff and t-tests and Mann–Whitney U tests were used to examine equivalency of survey outcomes from staff representing both models.

**Results:**

The 76 pharmacists who completed the training program survey rated the modules as “largely” meeting their learning needs. All five role-emergent pharmacists and 29 LTC participating staff reported positive experiences with the pharmacy preceptor-student-staff collaboration. Preceptors reported that having students work side-by-side with facility staff promoted inter-professional collaboration. The staff viewed students’ presence as a mutually beneficial experience, suggesting that the students’ presence had enabled them to deliver better care to the residents. As a direct result of the study findings, the annual role-emergent placement capacity was increased to over 45 by the end of the study.

**Conclusions:**

This study demonstrated that role-emergent LTC facilities were not only viable for quality institutional APPEs but also provided more available sites, greater student placement capacity, and more trained pharmacy preceptors than could be achieved in role-established facilities.

## Background

Accreditation standards in Canada and the US specify that schools of pharmacy must ensure that patient-centered and population-based care competencies serve as the basis for curriculum development and change. These standards also stipulate that experiential learning should embody the critical curriculum components to give students opportunities to practice and master these competencies [1-3].

In the Canadian province of British Columbia, the responsibility for designing and implementing experiential sites falls to the University of British Columbia’s (UBC) Faculty of Pharmaceutical Sciences as the sole post-secondary institution graduating pharmacists. At UBC, these experiential learning segments consist of three series of community pharmacy-based experiences: two 160-hour series of introductory pharmacy practice experiences (IPPE) completed at the end of the second and third years of the four-year curriculum and one 320-hour series of advanced pharmacy practice experiences (APPE), and an additional institutional-based 160-hour series of APPE delivered in the final year of the curriculum. This paper focuses on the school’s efforts to build greater placement capacity for its institutional-based APPE.

In keeping with traditional practice in pharmacy schools across North America, UBC places students into experiential learning segments only at sites with full-time on-site pharmacy preceptors who can provide continuous and direct student supervision. Placement sites with an on-site discipline specific preceptor to supervise and model the professional role to the student have typically been referred to in the literature as “role-established” sites [4,5]. In British Columbia, the only institutional settings offering continuous on-site pharmacist supervision were tertiary and community hospital settings -- mostly in acute care in-patient wards and some long-term care wards. Most other institutional settings such as outpatient clinics and stand-alone long-term care facilities had only limited pharmacy coverage, and as such were traditionally not considered to be eligible experiential sites for pharmacy students. However, over the past several years, numerous factors converged to make it increasingly challenging to continue to recruit and retain sufficient hospital sites to provide all UBC students with an institutional-based APPE. Contributing factors included: increased student enrolments, increased length of the institutional APPEs, increased emphasis on patient-centered activities and less on drug distribution services, shortages of hospital pharmacists, downsizing and amalgamation of institutional facilities, and increased expectations for hospital pharmacy departments to expand services such as: participating on ward-based and outpatient inter-professional teams to promote formulary policies and effective and safe medication use, routine allergy assessment and screening, review of medication charts, conducting discharge medication counselling, and implementing self-medication management programs for high risk patients prior to discharge. Similar challenges were also reported by other schools of pharmacy [1,6,7]. Under these circumstances, hospital pharmacists found it difficult to maintain their own employment and professional responsibilities while simultaneously assisting schools to provide students with patient care learning opportunities. This resulted in fewer hospitals willing to commit to precepting, making it difficult for the school to meet accreditation expectations requiring student exposure to institutional patient care practice. Consequently, the school has needed to consider alternate experiential models to meet this mandate.

With studies confirming the benefits of pharmacy-based patient care services to promote appropriate, safe and effective use of medications among elderly patients, expanding the institutional APPE sites to include Long-Term Care (LTC) facilities seemed a sensible solution to address capacity issues [8-14]. However, including LTC facilities as APPE sites remained problematic since the majority were serviced by off-site community pharmacists offering medication dispensing services remotely, with on-site time limited to once a week for patient care services. Experiential sites lacking on-site preceptors to supervise students have often been labelled in the allied health literature as “role-emergent”, “self directed”, “long-arm supervision”, “independent community placements”, or “non-traditional placement sites” [4,15,16]. Given the role-emergent nature of the LTC facilities, UBC’s pharmacy leadership, faculty members and prospective pharmacy preceptors were reluctant to fully endorse this non-traditional model without evidence it could provide comparable experience to that of the traditional role-established model. These questions were difficult to answer in the absence of any published reports discussing pharmacy preceptors’ and facility staff experiences within such settings.

This current demonstration study explored the viability of role-emergent LTC APPEs at sites which had traditionally not served as placement locations for pharmacy students. The specific objectives of this paper are to: (1) outline the support and training necessary for new preceptors, (2) discuss pharmacy preceptors’ and LTC non-pharmacist staff experiences with the new model, (3) compare their experiences with those at the more traditional role-established LTC APPE sites, and (4) document gains in numbers of available role-emergent sites as a consequence of the study’s findings. Project start-up costs were funded through the British Columbia Academic Health Council and included costs of additional support staff and program overhead to develop and deliver the preceptor training program and to design and evaluate the LTC role-emergent model.

## Methods

### Design

This demonstration study was designed as a comparative study to understand the experiences of both pharmacy preceptors and of non-pharmacy facility staff participating in the newly implemented role-emergent model versus the traditional role-established LTC APPE model. This study occurred over three academic years (August 2005 to May 2008) and consisted of three phases: (1) development phase between August to November 2005, (2) evaluation phase from January 2006 to May 2008, and (3) role-emergent placement expansion phase at the end of the study. Ethics approval was obtained from the Office of Research Services at UBC for research with “individuals whose data, or responses to interventions, stimuli or questions by a researcher are gathered or utilized for the purposes of a Research project” [17].

### Recruitment of LTC preceptors and facilities

Purposive sampling (where subjects are selected because of some distinguishing characteristic) was used to identify pharmacy preceptors and their affiliated LTC facility from a list that exemplified best LTC pharmacy practice models within British Columbia created by community and hospital pharmacy managers [18]. These facilities consisted of both role-established sites with on-site hospital pharmacists and role-emergent sites whose pharmacy requirements were contracted to a local community pharmacy. The first seven preceptors, two role-established and five role-emergent -- who agreed to participate were recruited. Table 1 summarizes the characteristics and staff composition of the LTC facilities recruited for this study. While the role-established pharmacists had previous histories of serving as preceptors for the traditional institutional APPEs, the community role-emergent pharmacists had no such experiences. The role-established preceptors participated in the development phase to define a LTC APPE syllabus based on their previous experiences and expertise. In this paper, “preceptor” means the pharmacy preceptor and all other LTC non-pharmacy personnel are referred to as “staff”. Since the aim of this study was to build institutional capacity using LTC role-emergent sites, all facilities served as evaluation sites to test the viability of this new model for pharmacy. As part of our school’s remuneration policy, all pharmacies received a stipend of $50.00 CDN for a four week student experience. All role-emergent LTC affiliated pharmacists and non-pharmacy staff participating in this study were assured their identity would remain confidential for the purpose of this study, and they were free to withdraw from the study at any time.

**Table 1:** Characteristics of long-term care facilities participating in the study (N=7)

	**Facility 1**	**Facility 2**	**Facility 3**	**Facility 4**	**Facility 5**	**Facility 6**	**Facility 7**
	**Role established**	**Role established**	**Role emergent**	**Role emergent**	**Role emergent**	**Role emergent**	**Role emergent**
**Affiliation**							
Hospital	**√**	**√**					
Community Pharmacy			**√**	**√**	**√**	**√**	**√**
**Ownership**							
Proprietary	**-**	**-**	**√**	**√**	**-**	**-**	**√**
Religious	**√**	**-**	**-**	**-**	**-**	**√**	**-**
Not for Profit	**√**	**-**	**-**	**-**	**-**	**-**	**-**
Government	**√**	**√**	**-**	**-**	**√**	**-**	**-**
**Level of Care**^†^	3	3	3	2	3	3	2
**Number of Beds**	221	150	38	103	80	200	115
**Ancillary Services**							
(Number) Type of Services*	(6) DT, Pod, OT, PT, ST, SW	(5) DT, OT, Pod, PT, SW	(3) DT, Pod, PT	(4) PT, DT, Pod, OT	(3) DT, Pod, PT	(5) DT, OT, PT, ST, SW	(3) DT, Pod, PT
(Number) Type of non-pharmacy students*	(5) DMD, DT, MD, OT, PT	0	0	(1) Aide	0	(3) DT, RN, OT	(2) Aide, RN
**Care provided by resident’s physician**	**√**	**√**	**√**	**√**	**√**	**√**	**√**
**Care provided by house physician**	**-**	**-**	**-**	**-**	**-**	**-**	**√**
**Patient Care Conferences**	**√**	**√**	**√**	**√**	**√**	**√**	**√**
**Resident’s Chart Available to Pharmacists for Documentation**	**√**	**√**	**√**	**-**	**-**	**√**	Separate Care plan inserted

*RN Registered Nurse, *Aide* Nurse’s Aide, DMD=Dentistry, *DT* Dietician, *MD* Medicine, *OT* Occupational Therapist, *Pod* Podiatrist, *PT* Physical Therapist, *ST* Speech Therapist, *SW* Social Worker.

^†^Level 3 care: clients need 24 hour professional nursing care and supervision, medical and therapeutic care in required, and a minimum 2.5 hours of personal care is needed. Level 2 care: clients need 24 hour of personal care, medical and, or nursing care is required, individual care may range from 1.2-2.5 hours per day.

### Preparation: training program for potential LTC preceptors

Since the APPEs for role-emergent LTC locations were expected to be less structured - thus requiring students to be more self-directed, preceptor preparation was essential to ensure the APPE’s learning outcomes were met [4,15,16]. An education program was introduced to help preceptors acquire basic knowledge and skills pertaining to care of elderly patients. Those invited to the program included all pharmacists who were affiliated with a LTC site (emergent or established) and interested in enhancing their competence for providing care to senior patients and in increasing their confidence to mentor students within such a practice. While the school did not require an immediate commitment from the pharmacists to take a LTC APPE pharmacy student, the invitation was extended to all those who were open to exploring the possibility of creating future LTC APPE placement within their affiliated sites. The program was initially piloted with LTC APPE preceptors participating in the first cycle of the evaluation phase (January – May 2006) and was delivered over one-half day. The program featured a case-based approach using lectures, small group discussions, and exercises requiring pharmacists to identify drug and non-drug related issues and to develop care plans for these issues. During subsequent cycles (September 2006-May 2008), the program was extended to a full day and was open to all community and institutional APPE preceptors who were interested in developing future APPE content specific to the care of older adults in their practices. A Jeopardy®-like game and an expert panel of inter-professional health providers from pharmacy, occupational therapy, physiotherapy and nursing was introduced to promote a more interactive delivery of the case content. The case was built on an existing “Care of Elders Delirium Module” developed at the University of British Columbia for promoting inter-professional collaboration [19]. The module facilitated learning about commonly encountered conditions among elderly patients in LTC facilities including: delirium, depression, dementia, urinary incontinence, urinary tract infection, anemia, pain control, chronic heart failure, atrial fibrillation, stroke, renal failure and alcohol withdrawal. The case also allowed participants to deal with implications of physiological changes in the aged, and provided an overview of common geriatric assessment tools. Writers and reviewers with advanced clinical training were retained to develop a participant’s educational tool kit which included completed care plans for all drug-related issues within the case and evidence-based summaries for the management for all drug related issues that arose in the case. Because of the scarcity of geriatric pharmacology textbooks, the kit was supplemented with learning resources from the primary and secondary literature.

### LTC preceptor support

Consistent with the school’s other APPE program, both LTC APPE students and preceptors recruited for the evaluation phase received the same 150-page APPE manual, which includes a list of learning activities that is specific to the APPE setting. The manual is organized into several sections intended to facilitate teaching and learning process during APPEs. The first section provided an overview of the APPE, a list of expected competencies and outcome-based objectives so that the students and preceptors understood the expectations of the APPE, a list of learning activities to meet the intended objectives and a week-by-week activity calendar proposing how the student learning activities could be distributed throughout the weeks allotted to the APPE. For the LTC APPE, the list of learning activities is outlined in Table 2. The subsequent sections of the manual served as a resource to guide students and preceptors through the APPE and included: an orientation checklist to be followed when new students came to the site; a policy and procedure section that outlined rules on attendance, attire, preparedness, professionalism; an evaluation form and evaluation procedure detailing the components to be considered for the final grade; and patient care documentation tools to facilitate delivery of care and to guide discussions between preceptors and students. The school’s APPE faculty met with the preceptors to discuss the content of the manual, and all preceptors were supported by the school through site-visits, telephone communication, and written e-mail/ webmail communications on an as-needed basis. Contact with preceptors was made on average once a week.

**Table 2:** Student learning activities at the long-term care facilities (4-week experience)

**Learning activities**	**Minimums**
**1. Provide Comprehensive pharmaceutical care:**	3-5 patients over 4-weeks
● Assess for drug-related problems (DRPs)	
● Identify and list all actual and potential DRPs	
● Create an initial care plan for each DRPs and discuss with pharmacy preceptor	
● Collaborate with physician/ facility staff and, or patient to resolve or prevent the DRPs.	
● Provide follow-up to all patients	
**2. Participate in Drug Review Process:**	2-8 hours over 4-weeks
● Participate in daily drug review process by reviewing patients’ medication profiles on designated unit	
● Assess for DRPs	
● Identify and list all actual and potential DRPs	
● Create an initial care plan for each DRPs and discuss with pharmacy preceptor	
● Collaborate with physician/ facility staff and, or patient to resolve or prevent the DRPs.	
**3. Conduct Allergy Assessments:**	4-5 patients over 4-weeks
● Assess patients for drug allergies	
● Discuss allergies with pharmacy preceptor	
● Discuss allergies with physician/ facility staff	
● Document all allergies on the form provided	
**4. Provide Medication Teaching:**	4-5 patients over 4-weeks
● Provide medication teaching to patients and their families	
**5. Provide Presentations to the facility staff:**	1 of each type of presentation over 4-weeks
● On a patient case to which comprehensive pharmaceutical care has been provided	
● On a topic of interest	
**6. Conduct Critical Appraisal of the Literature:**	1 critical review over 4-weeks
● Critically review one article relevant to care of your patients and discuss with pharmacy preceptor	
● Document the appraisal on the form provided	
**7. Provide Drug Information:**	3 drug information workups over 4-weeks
● Work-up patient specific drug information questions raised by staff at the facility	
● Document all drug information questions on the form provided	
**8. Engage in Inter-professional Collaboration:**	Daily and on-going
● Participate in patient care-conferences	
**9. Participate in Drug Distribution at the long-term care facility:**	Daily and on-going
● Discuss drug distribution system with pharmacy preceptor	
● Observe Nurse and Unit Clerk in distribution process	
● Participate in processing and clarification of medication orders at the designated unit	
**10. Understand Medication Management Processes and Protocols at the facility:**	No minimum, as time permitting
● Discuss programs at the institution to improve the quality of drug use, for example: drug utilization programs, adverse drug reporting protocols, etc.	
● Discuss formulary system	
**11. Participate in other activities identified as appropriate by pharmacy preceptor**	No minimum, as time permitting

To ensure adequate learning support for students, the school proposed all preceptors schedule a one-day orientation session at the start of the APPE to introduce their student to the facility staff, to provide students with a tour of the facility and its programs, and to provide the staff with an overview of the students’ learning activities. Preceptors also scheduled regular face-to-face meeting times with their students for at least one-half day per week to monitor students’ progress, to provide direct supervision, and to carry out formal assessments of the student’s findings. Preceptors were available to students at all times via telephone and email to discuss students’ proposed patient care interventions and drug information responses, before they were disseminated to health care team members.

### Data collection

At the end of the educational program, pharmacists were asked to report using a 15-question survey - how well the Care for Elders Delirium Module had met their own learning needs on a 5-point scale (5=Totally, 4=Largely, 3=Fairly, 2=Poorly, 1=Not at all). Those pharmacists who participated in the role-emergent APPE also participated in a follow-up telephone interview to obtain their viewpoints on the APPE. Similarly, the non-pharmacy facility staff was invited to share their experiences using a seven question survey that required selecting responses from predefined 4-point scale: (4=Very, 3=Somewhat, 2=Only a little, 1=Not at all). Additionally, staff were encouraged to share their thoughts using open-ended response formats in the spaces provided the end of the survey. Pharmacy preceptors handed out the surveys to staff at their facilities on one of their on-site visits and instructed them to deposit the completed survey in a receptacle box. Staff were assured that their responses would be delivered directly to the research office without being read by the site pharmacists and the survey return boxes were labelled accordingly. UBC’s Office of Research Services permits research data to be collected only from research participants who provide written Informed Consent to allow use of their data by the study team.

### Analysis

Preceptors and LTC facility staff reports of their perceptions and experiences were transferred onto a spreadsheet (Microsoft Excel 2000) then uploaded to SPSS Ver. 18 (IBM, 2011). Descriptive statistics (frequencies, means and standard deviations) on the LTC facilities characteristics and survey questions were computed and quantitatively analyzed. Differences of opinion between staff in role-established versus role-emergent facilities were examined via t-tests with significance set at p<.05 and confirmed (for ordinal data) with Mann–Whitney U tests. Free-form and open-ended responses were categorized thematically and analyzed. All identifiers were removed to maintain anonymity of participants.

## Results

In all, 88 pharmacists participated in the training program, 7 pharmacists (n=2 role-established and n=5 role-emergent) were recruited to serve as LTC preceptors for the evaluation phase and 29 non-pharmacy LTC staff participated in the demonstration study. We report results from this study in five sections; (1) pharmacists’ experiences with the education program, (2) characteristics of the facilities and preceptors where the APPEs were located, (3) pharmacy preceptors’ experiences during five cycles of the APPE itself, (4) experiences of non-pharmacy facility staff in working with the APPE, and (5) gains in numbers of role-emergent placements at the end of the study.

### Pharmacists’ experience with the education program

Eighty six percent (n=76) of pharmacists completed the 15-question survey on how well the Care for Elders Delirium Module had met their own learning. Given the anonymity of the surveys, the data could not be categorized into those who participated in the evaluation phase versus those who remained potential recruits. Respondents assessed the module as “largely” meeting their own learning needs (4.3 out of 5) and reported the Jeopardy® Game to be equally “largely” effective for learning (4.5 out of 5), 75% found the content to be relevant to their practice, 87% indicated the case embodied problems and issues typically encountered in practice, and 57% reported they had gained a better understanding of the roles and responsibilities of the different health professionals within residential care settings (Table 3). The most frequently cited new knowledge included: the differences between delirium, depression and dementia; relevance of laboratory values; the roles of other health care professionals; the relevance of physiological and pharmacokinetic parameters in managing drug therapy; and strategies to develop and implement care plans. The one-day education program was delivered two months prior to beginning actual APPE rotations in January 2006, and was offered twice: once on a weekday and then again on a weekend to accommodate pharmacists’ work schedules.

**Table 3:** Pharmacists’ learning needs met by the care of elders module (N=76)

Questions^*^		**Mean ± SD**
1. The material provided was presented in an understandable manner.		4.4 ± 0.7
2. The program has met the stated learning objectives effectively.		4.4 ± 0.7
3. The jeopardy game was helpful and effective for learning		4.3 ± 0.8
4. How realistic and true-to-life was the case?		4.3 ± 0.8
5. Did the case embody problems and issues typically found in actual practice?		4.2 ± 0.8
6. Did the case complexity or difficulty level challenge you?		4.1 ± 0.9
7. Is the case complexity and difficult level appropriate for entry-level health personnel?		3.9 ± 0.8
8. Did you have sufficient knowledge from your own previous experience?		3.7 ± 0.8
9. How much new information about health/medical issues did you learn?		3.9 ± 0.8
10. How much did you learn about different professional roles and responsibilities in interdisciplinary settings?		3.9 ± 0.9
11. Did the panel provide new, critical information as needed?		4.2 ± 0.7
12. How much did you learn that is relevant to your own practice?		4.0 ± 0.8
13. Did the facilitator help your group to develop relevant pharmacy care plans?		4.5 ± 0.6
14. The jeopardy game was helpful and effective for learning		4.3 ± 0.7
15. **Overall Assessment on a 1 to 5 scale**		4.3 ± 0.7
16. Please suggest two or three things you learned today that were new information to you	● Delirium vs. Dementia vs Depression	
	● Interpreting laboratory values in seniors	
	● Role of other Health Professions in senior care	
	● Physiology and pharmacokinetic considerations that need to be made re – drug therapy in seniors	
	● Process for developing care plans	
17. Please name two to three changes in your own practice that you will implement as a result of what you learned:	● Incorporate comprehensive patient care process for seniors, such as thorough assessment and monitoring	
	● Incorporate inter-professional collaboration and referral processes	
	● Will be able to better support students	

^*^Response Scale: 1=Not at all, 2=Poorly Met, 3=Fairly Met, 4=Largely Met, 5=Totally Met.

### Characteristics of role-emergent and role-established LTC APPE facilities

The seven LTC facilities participating in the evaluation phase – two role-established and five role-emergent, together created 23 LTC APPE placements from January 2006 to May 2008. One placement represents one student. While the ownership and the ancillary services differed across facilities; with the exception of one facility, all residents residing in these LTC facilities had access to a physiotherapist, dietician, podiatrist, nurse and medical care provided by an off-site primary care physician (Table 1). The resident’s chart served as an important tool for pharmacists to document their care at all but two role-emergent LTC facilities. The single key difference between the role-established and role-emergent LTC facilities was the availability of an on-site pharmacy preceptor. At the role-emergent sites, students and preceptors had to schedule regular meetings to discuss learning and care activities. On days when the role-emergent preceptor was absent, the allied health and nursing staff played an instrumental role in providing student oversight.

### Role-emergent APPE pharmacy preceptors’ experiences

Overall, preceptors expressed positive experiences with the APPE. Post-APPE follow-up interview data revealed that preceptors believed the students had learned substantially from this experience and confirmed participation in future LTC APPEs. Their comments included such observations as *“Just wanted to say how great it has been to have the student … (student) is an awesome resource for the nurses and a help to me in following up on things I wish I was there to do.”* Another preceptor said *“I think (the student) is getting an amazing learning experience - interacting with patients, nurses and physicians and problem solving. (The student) has helped to resolve some nursing issues … helped managed an insulin start … conducted an in-service to the nurses … attended a medication safety advisory committee meeting and a nursing medication incident meeting … I don't think she has had a boring day yet!,* while a third said *“It might be too soon to say this, but I wish I could have a student at each of my facilities!”* A particularly summative comment came from a role-emergent preceptor who said *“I think this was the best facility for a pharmacy student … I hope the next batch of students will have as rewarding an experience.”*

Some preceptors did note that their LTC site offered students limited exposure to certain traditional hospital learning activities such as conducting allergy assessments and providing one-on-one patient education. However, generally all expressed that other opportunities unique to LTC - such as working side-by-side with the facility staff to enhance residents’ care and access to extensive medication and health record to review and assess for drug related issues, made up for these deficiencies.

### Non-pharmacy facility staff experiences at role-emergent and role-established APPE

Additional post-APPE surveys were completed by 29 staff: 8 at role-established and 21 at role-emergent sites (Table 4), and consisted primarily of registered nurses, care aides and licensed practical nurses. Non-pharmacy staff reported overall positive experiences (Table 5). There were no statistically significant differences between staff experiences in role-established versus role-emergent facilities on any of the 7 features although there were minor variations: staff at role-emergent sites were slightly more familiar with the students’ purpose at their site, knew how to refer patients to students, found student services to residents to be helpful, developed their own better understandings of the pharmacist role, and found students to have provided helpful services at the placement site. In contrast, staff at role-established sites reported fractionally more opportunities for student inter-collaboration and more professional communications and interactions with residents. Across all sites, more than half of the respondents remarked that even though they were initially uncertain of how to refer residential patients to the students, they made deliberate effort to engage students in the patients’ care; and 64% indicated the services provided by the students were very helpful to the patients. A similar 64% also reported that students’ services benefitted them directly and 25% suggested they now had a better understanding of the role of the pharmacist at their facility as a consequence of working with the students. Three staff from different institutions spoke about the benefits of pharmacy students at the role-emergent facilities: *“If we have questions about medications, they are available right away. We can collaborate with them. By listening to them (pharmacy students) teaching a patient, we were able to learn from them.”* Another said, *“When a new order came around I was unfamiliar with, I found the pharmacy student very knowledgeable of said drugs … indications, etc.… plus when dealing with pharmacy she (the student) helped the process out by providing information in dealing with them (community pharmacy staff), questions they would ask to make the process smoother.”* Staff also recognized that successful APPEs result not only in student learning but also in improved care for residents, *“She (the student) helped us in reducing loxapine 10 mg with our resident by suggesting to the doctor the tapering process, thus reducing dyskinetic effects of loxapine. Good job …!”*

**Table 4:** Characteristics of staff survey respondents (N=29)

	**Facility 1**	**Facility 2**	**Facility 3**	**Facility 4**	**Facility 5**	**Facility 6**	**Facility 7**
	**Role established**	**Role established**	**Role emergent**	**Role emergent**	**Role emergent**	**Role emergent**	**Role emergent**
**Distribution**							
Registered Nurse (RN)	3 RNs	1 RN	2 RNs	2 RNs	2 RNs	3 RNs	-
Nurse’s Aide (Aide)	-	-	4 Aides	1 Aide	-	-	-
Licenced Practical Nurses (LPN)	-	1 LPN	1 LPN	-	-	-	5 LPNs
Other	2 Pastoral Care; 1 Dietician	-	1 (not specified)	-	1 Administrator	-	-
**Shifts Worked**							
Days	2 RNs; 2 Pastoral Care; 1 Dietician	1 RN; 1 LPN	5 RNs	1 RN	2 RNs; 1 Administrator	3 RNs	2 LPNs
Evenings	-	-	2 RNs	-	-	-	-
Graveyards	-	-	-	-	-	-	-
Mixtures	1 RN	-	-	2 RN; 1 Aide	-	-	3 LPNs
**Employment Status**							
Full-time	3 RNs; 2 Pastoral Care; 1 Dietician		7 RNs	1 RN	2 RNs; 1 Administrator	1 RN	3 LPNs
Part-time		1 RN; 1 LPN		1 RN		2 RNs	
Casual				1 Aide			2 LPNs

**Table 5:** Responses to the staff survey (N=29)

Staff responses*	**Role-established ** **facilities (n=8)**		**Role-emergent facilities (n=21)**		**Significance of the difference**	
**Number of respondents**	**Mean±SD**	**Median**	**Mean±SD**	**Median**	t-test**	MWW**
How familiar were you with the purpose of the students at your setting?	3.13 ± 0.64	3	3.29 ± 0.78	3	ns	ns
Did you know how to refer residents to the student?	2.74 ± 1.16	3	3.24 ± 0.94	3	ns	ns
Were the students professional in their communications and interactions with the residents?	3.86 ± 0.38	4	3.75 ± 0.55	4	ns	ns
Were the services provided by students helpful to residents?	3.57 ± 0.54	4	3.67 ± 0.48	4	ns	ns
Are there opportunities for student to collaborate with other students at your LTC facility?	3.43 ± 0.54	3	3.34 ± 0.82	4	ns	ns
By interacting with the students, did you develop a better understanding of the pharmacist’s role?	3.25 ± 1.04	4	3.38 ± 0.67	3	ns	ns
Did you find the services provided by the students helpful to you?	3.43 ± 0.79	4	3.57 ± 0.60	4	ns	ns

^*^Response Scale: 1=Not at all; 2=Only a little; 3=Somewhat; 4=Very.

**Significance set at p<.05 and confirmed (for ordinal data) with Mann–Whitney U (MWW).

There was overall consensus by both pharmacy preceptors and facility staff that successful institutional APPEs could be located in role-emergent LTC settings, with equal effectiveness as in role-established settings and with additional benefits of exposure to a growing sector of health services to the elderly.

### Gains in numbers of role-emergent placements at the end of the study

**Figure 1:**
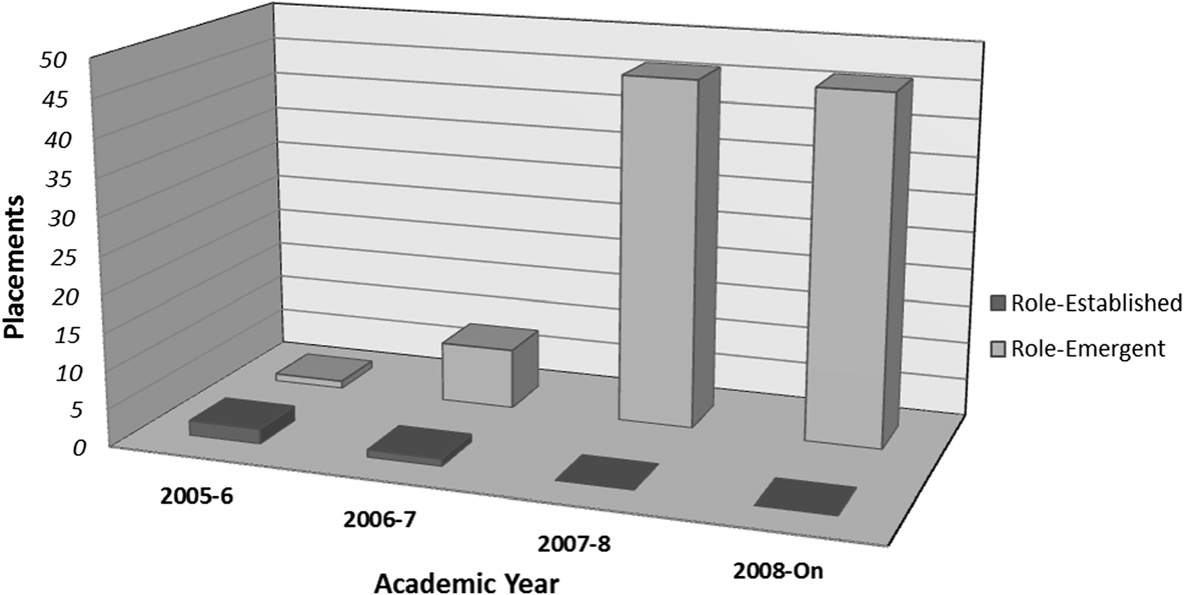
LTC placements capacity over four-academic years for role-emergent versus role-established sites.

As a direct consequence of this study, the school was able to expand its institutional-based APPE placement capacity by recruiting from the already trained pool of preceptors. Figure 1 shows that opportunity for growth and expansion of LTC placements was much more evident at role-emergent sites than at role-established ones. This was not surprising since the majority of the newly trained preceptors were affiliated with role-emergent than role-established facilities. Together, the role-emergent preceptors represented about a dozen different LTC establishments, some single-site but many multi-site. The numbers of placement per LTC establishment ranged from one to 12 students, spread over the academic year (from September through April). Together, this increased annual student placement capacity at the role-emergent LTC facilities from zero at the study’s outset to a total of 46 by the end of the study.

## Discussion

It has long been recognized that the healthcare needs of seniors residing in LTC facilities are complex and require the attention of a variety of healthcare providers. Several studies have confirmed that the presence of pharmacist within these facilities contributes to improved drug therapy management [8-13]. Academic-based studies have also demonstrated that pharmacy students under the direct supervision of pharmacy preceptors contribute effectively to patient care during their practice education experiences [20-22]. However, experiences involving pharmacy students within role-emergent settings have been lacking, and findings from this study provide some understanding to this end. This study demonstrated that role-emergent LTC facilities are a viable option for institutional APPEs. Within these settings, inter-professional collaboration naturally manifested itself with many nursing staff taking on the role of surrogate preceptors while the pharmacy preceptor was off-site. In this paper, the term “preceptor” is reserved for the pharmacy preceptor and LTC facility staff referred to as “staff”. The pharmacy preceptors communicated routinely with the LTC staff both during their weekly visits to the facilities and by phone as necessary. Student supervision was the responsibility of these pharmacy preceptors, but with occasional input from facility staff. Direct pharmacy preceptor-student-staff collaborations became integral to supporting both pharmacy preceptors in their efforts to provide students with relevant patient care opportunities and to the nursing staff in their efforts to provide optimal patient care.

Reviewing the staff surveys, it was apparent that most health care providers viewed students’ presence at the LTC facilities as a mutually beneficial experience. Many felt that they delivered better care to residents as a result of the services provided by the students to the residents and to themselves. A large number of staff indicated they had developed a better understanding of the pharmacist’s role through their interaction with the pharmacy students. When asked what changes they might suggest to the APPE activities, the staff suggested: participating in regular bed-side medication rounds with the staff, setting aside more time to serve as resource for the nursing staff, increasing their involvement with seniors who have questions about their health/medications, and providing education to nursing staff on topics related to medications and their safe administration. Some staff also suggested that future students should occasionally work the night shifts to enhance their learning and to support nurses during that shift. Hence, overall, the students’ presence and their engagement in APPE activities contributed towards shaping and expanding the pharmacist’s role at these LTC facilities. Others have shared similar reciprocal staff-student learning within an inter-professional experiential environment [5,23]*.*

The time committed by the school at the beginning of the study to support preceptors through discussions and workshops, and preceptors’ efforts to orient the students to the facility and its staff prior to the start of the APPE were likely important factors contributing to the success of these experiences. In their feedback, preceptors noted that the educational support extended to them had increased their awareness of how to structure student learning within their LTC facilities. Moreover, the opportunity to interact with the inter-professional panel during the Care of Elders Delirium Module workshop had provided them with useful strategies for promoting inter-professional collaboration at their sites, which manifested in a rich learning environment for their students. Examples of some of these activities include: shadowing nurses during medication administration, providing in-services for the staff on relevant medication issues, providing information on questions about patients’ medications, and assisting with medication orders that required clarification. Preceptors also reported learning new knowledge, skills and processes by participating in the Care of Elders Delirium Module, which they believed would help them guide their students. Additionally, as part of the APPE expectations, all placements began with a tour of the facility provided by the preceptor where students were introduced to most of the staff and programs available at the LTC. Preceptors and students met face-to-face at least one-half day per week during which time preceptors provided direct supervision and carried out formal assessments of the student’s findings, care plans, and recommendations. Written care plans, drug information responses and consultation notes to the physician were emailed to the preceptor for feedback prior to dissemination. Preceptors were available to students at all times via telephone and email to discuss proposed interventions, before they were discussed with health care team members. Hence, despite the seemingly unstructured nature of these role-emergent APPE placements, there was sufficient structure for checks and balances to ensure appropriate, effective and safe care by students.

### Limitations

Like any study, certain limitations still exist. The first is the small sample size of staff who responded to the survey. Given that this was a demonstration study and that the sample represented the majority of the disciplines at the LTC facilities, feedback provided valuable insight on the impact of the APPE on staff. However, future studies should aim to validate the findings with other disciplines not represented here. Second, role-emergent placements were limited to senior year pharmacy student APPEs. These students had previously completed two placement experiences – one in their second year and another in their third year. It remains untested whether such experiential models offered earlier in students’ academic programs would have the same success.

## Conclusion

The analysis of pharmacy preceptors’ and facility staff data has provided a greater understanding of the role-emergent experiential model in pharmacy education. Within the context of our school, the role-emergent model provided a beneficial experience for both preceptors and staff. Prior to this study, there had been no exposure of pharmacy students to role-emergent LTC facilities, and this model was seen as an attempt to fill a shortage of role-established institutional sites. However, the study results have shifted this view, and now such sites are being recognized as offering legitimate institutional-based learning experiences [4]. We hope these findings will serve as a catalyst for greater adoption of role-emergent approaches and will stimulate further research to examine whether similar successes can be achieved within different contexts.

Based on the pharmacy preceptors’ and facility staff’s positive experiences, two of the participating role-emergent sites offered to increase their original student placement number from one to five and six students per academic year, respectively. In addition, the school confirmed two new role-emergent LTC facilities at the end of the study for subsequent academic years, and registered six other facilities as potential future placement sites. Together, this increased student placement capacity at the role-emergent LTC facilities from none to over 45 by the end of the study - a number well exceeding expectations.

## Abbreviations

APPE: Advanced pharmacy practice Experience; IPPE: Introductory pharmacy practice Experience; LTC: Long-term care; UBC: University of British Columbia.

## Competing interests

The authors report no conflicts of interest in this work. The authors meet the criteria for authorship as recommended by the International Committee of Medical Journal Editors. John B. Collins received consultancy fees for analysis and interpretation of the study data. The authors received no compensation related to the development of the manuscript.

## Authors’ contributions

RK conceived the design of the study, implemented the study, and interpreted the data. MK participated in the design, implementation, collation and data analysis of the study, and JBC participated in the analysis and interpretation of the data. All authors contributed to drafting the manuscript and read and approved the final version of the manuscript.

## Pre-publication history

The pre-publication history for this paper can be accessed here:

http://www.biomedcentral.com/1472-6920/13/104/prepub
